# Pyroelectricity in poled all-organic polar polynorbornene/polydimethylsiloxane-based stretchable electrets

**DOI:** 10.1039/d4tc00791c

**Published:** 2024-05-16

**Authors:** Thulasinath Raman Venkatesan, Francis Owusu, Frank A. Nüesch, Manuel Schulze, Dorina M. Opris

**Affiliations:** a Laboratory for Functional Polymers, EMPA Swiss Federal Laboratories for Materials Science and Technology Überlandstrasse 129 8600 Dübendorf Switzerland Thulasinath.ramanvenkatesan@empa.ch; b Institute of Chemical Sciences and Engineering, Ecole Polytechnique Federale de Lausanne (EPFL) Station 6 1015 Lausanne Switzerland; c Institute of Materials Science and Engineering, Ecole Polytechnique Federale de Lausanne (EPFL) Station 6 1015 Lausanne Switzerland; d Institute of Physics and Astronomy, University of Potsdam Karl-Liebknecht-strasse 24/25 14476 Potsdam Germany; e Departments of Materials, ETH Zürich Vladimir-Prelog-Weg 5 8093 Zürich Switzerland

## Abstract

Pyroelectricity in a recently developed all-organic composite electret with a polar polynorbornene-based filler and polydimethylsiloxane (PDMS) matrix has been studied with the help of thermal and dielectric techniques. Measurement of the pyroelectric *p* coefficient using a quasi-static periodic temperature variation at RT shows a non-linear dependence with the applied poling field, which is uncharacteristic of amorphous materials. Dielectric relaxation spectroscopy (DRS) and the thermally stimulated depolarization current (TSDC) technique reveal that this behaviour can be attributed to Maxwell–Wagner interface (MWI) polarization that occurs at the filler–matrix interface. These charges released during the onset of dipolar *α* and *β* relaxations of the filler particles contribute majorly to the observed pyroelectricity at RT. The saturation of both MWI TSDC shoulders and spontaneous polarization at higher electric fields correlates with the *p* coefficient value reaching a plateau at these applied fields. A maximum *p* coefficient of 0.54 μC m^−2^ K^−1^ is calculated for a poling field of 30 V μm^−1^.

## Abstract 2

A novel all-organic polar elastic composite consisting of a polar polynorbornene-based filler in a PDMS matrix shows room temperature non-linear pyroelectricity due to Maxwell–Wagner interfacial polarization and secondary pyroelectricity.

## Graphical abstract

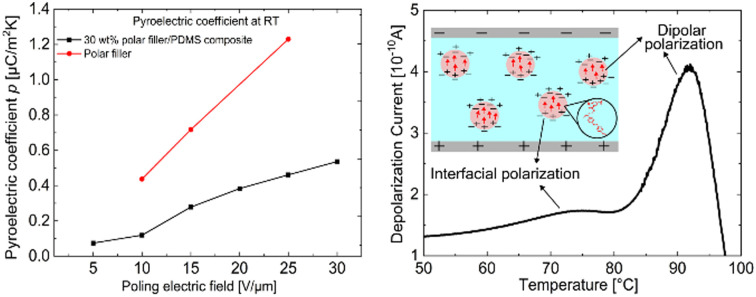

## 1 Introduction

Electric charges have been a source of fascination and fantasy to humankind for several thousand years. The earliest recorded description of electric charges spans about 2400 years ago when the Greek philosopher Theophrastus wrote about the property of lyngourion (believed to be tourmaline) of attracting particles from fire and pieces of wood.^1,2^ Though the emphasis of this description focused more on the mythical origin and supposed medicinal properties of this material, it was essentially the observation of electrostatic effects that occur due to temperature changes in the crystals, *i.e.*, the pyroelectric effect. A more scientific depiction of the attraction and repulsion of electric charges by materials such as amber was done by Thales of Miletus, a Greek merchant and philosopher (∼624 B.C. to ∼546 B.C.).^3^ In the 18th and 19th centuries, after two millennia, much scientific curiosity arose in the field of electrostatics as several philosophers throughout Europe studied various natural materials that exhibited electric charge-related phenomena.^1,3^ This includes the first scientific description of pyroelectricity (pyr, Greek word for ‘fire’) in a scientific journal by Louis Lenery, a physician and chemist in 1717, and the discovery of piezoelectricity (piezo, Greek word ‘to press’) by the Curie brothers in 1880.^1,4^ Furthermore, ferroelectricity (ferro, Greek word for ‘iron’, due to the similarity to magnetic properties) in Rochelle salts was reported by Valasek in 1971.^5^ This class of materials exhibiting a quasi-permanent dipolar polarization is referred to as electrets.^6,7^

All these aforementioned electro-active properties are exhibited by crystalline materials with a non-centrosymmetric structure. Piezoelectricity encompasses materials that can produce an electrical output for a mechanical input. Pyroelectricity is a sub-property of piezoelectricity where a variation in temperature produces a reversible electrical signal. Ferroelectricity is again a sub-property of pyroelectric materials whereby the polarization direction in the electrets can be switched by changing the electric field direction. Hence, all ferroelectric materials by default are pyro- and piezo-electric; however, the reverse might not be true. For example, while polyvinylidene fluoride (PVDF) exhibits ferro-, pyro- and piezoelectric properties, quartz shows only piezoelectric properties, but not pyro- and ferro-electricity. Piezoelectricity can be described by a charge-spring model put forward by Gerhard.^8^ The model assumes that dipolar charges connected to springs placed between electrodes and the difference in mechanical and electrical characteristics of the respective charges and springs lead to the observed electro-active property.

In the 1980s, cellular polypropylene films were developed, which were later found to exhibit properties similar to crystalline and semi-crystalline electret materials, leading to a new class of electrets known as ferro- or piezo-electrets. Along with the subsequent development of dielectric elastomers (electro-electrets, EE) the definition of electrets was extended to also include amorphous materials.^7^ In the case of amorphous polymers, they can be cross-linked to make them stretchable, in addition to the flexibility offered by polymers. Recently, Opris *et al.* have developed all-organic elastomer electrets containing polar filler particles in a stretchable polydimethylsiloxane (PDMS) matrix, which can show high permittivity above the glass transition (*T*_g_) of the filler particles.^9–11^ Especially, the elastomer composite containing 30 wt% of polynorbornene (PNBE) filler particles modified with disperse-red dye (DR1) shows a very high piezoelectric *d*_31_ constant of 37 pC N^−1^.^9^

The pyroelectric effect can be used in several applications such as sensors, thermal imaging and energy harvesting.^12–14^ Especially, the pyroelectric properties of such novel amorphous composites are interesting because, as included in the review of Zhang *et al*.,^13^ the majority of pyroelectric materials are based on ceramics and single crystals, which lack flexibility and pose difficulties of being used over large surface areas. In the case of PVDF and its co-polymers—the commonly used ferroelectric polymers—the presence of fluoride makes them environmentally unfriendly and leads to various health concerns. Especially, the recent proposal of the European Commission to ban the production and use of all per- and polyfluoroalkyl substances has urged the need to find alternative materials that could replace fluorinated polymers.^15^ Hence, in this work, the pyroelectric properties of a novel PNBE-DR1 filled PDMS composite were investigated in detail using DSC and dielectric techniques. In addition, thermally stimulated depolarization current (TSDC) experiments have been performed with the aim of discriminating the dipolar charges from real charges and estimating the polarization of these composites.

## 2 Materials and methods

### 2.1 Synthesis and sample preparation

The detailed procedure for the synthesis of 5-norbornene-2-carboxylic acid (pure exo) and its subsequent functionalization with *N*-ethyl-*N*-(2-hydroxyethyl)-4-(4-nitrophenylazo) aniline (DR1), and preparation of free-standing composite films are reported elsewhere.^9,10^

### 2.2 Poling

To make the composite electro-active, the sample was subjected to a poling procedure as follows: the sample was initially heated to 110 °C, at the poling temperature (*T*_p_), above the *T*_g_ of filler particles. This was followed by applying an electric field (*E*_p_) to orient the dipoles in the filler for 10 min (*t*_p_). Subsequently, the composite film was cooled to 0 °C under the applied field to freeze the oriented dipoles of the filler. The DC poling voltage was applied using a Stanford Research Systems PS350 high-voltage source *via* contact metal electrodes to the sample heated to the poling temperature inside a Climatic Temperature System test chamber.

### 2.3 Differential scanning calorimetry (DSC)

DSC measurements were performed using a PerkinElmer DSC 8000 calorimeter. The sample was initially heated to 200 °C to remove the thermal history. This was followed by a cooling and heating cycle from 200 °C to −50 °C and then back to 200 °C. A heating/cooling rate of 20 K min^−1^ was used.

### 2.4 Dielectric relaxation spectroscopy (DRS)

A Novocontrol Alpha-A frequency analyzer was used to supply a voltage of 1 V for the DRS measurements carried out between 10^−1^ Hz and 10^+6^ Hz. A Novocontrol Quatro cryosystem was used to control the sample temperature, which was varied between −30 °C and +150 °C with a 2.5 K temperature step under a dry nitrogen atmosphere.

### 2.5 Thermally stimulated depolarization current (TSDC) technique

A Keysight B2985A electrometer with a built-in DC voltage source was used to both pole and measure the depolarization currents from the sample film. The composite film was poled at a temperature of 110 °C for 10 min before cooling it to 0 °C under the applied field before the start of TSDC measurements. The films were heated at 5 K min^−1^ using a Novocontrol Quatro cryosystem under a dry nitrogen atmosphere.

### 2.6 Pyroelectric measurements

Pyroelectricity can be defined as the reversible change in polarization that occurs due to a temperature change in a material under constant stress. It is measured using the pyroelectric coefficient *p* and given by:1
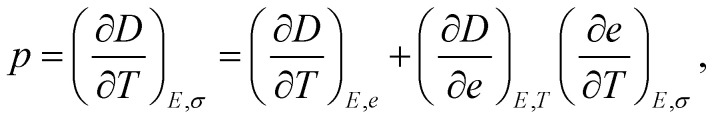
where *D* = dielectric displacement, *T* = temperature, *σ* = stress, *e* = strain and *E* = electric field. As seen from eqn (1), the pyroelectric coefficient has two components. The first term (∂*D*/∂*T*)_*E*,*e*_ denotes the primary pyroelectric effect caused by the change in polarization of the sample due to the temperature variation when the dimensions of the sample are constant. The second term can be expressed as:^16,17^2
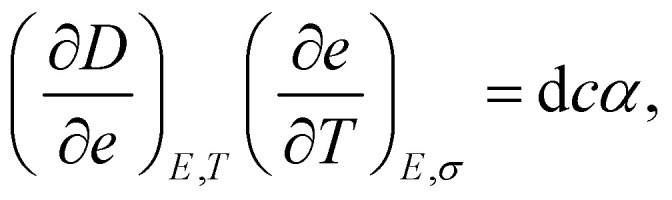
where *d* = piezoelectric coefficient, *c* = elastic stiffness and *α* = coefficient of thermal expansion. Eqn (2) represents the secondary pyroelectric effect due to piezoelectrically induced charges as a result of thermal expansion of the dielectric on heating.

To measure the pyroelectric coefficient in this work, a quasi-static periodic sinusoidal temperature variation was applied to a poled dielectric composite film using a Novocontrol Quatro cryosystem. A modulation frequency of 8.3 mHz, a mean temperature amplitude of 1 K and a mean temperature of 25 °C were used for the measurements. The resulting current was measured using a Keysight B2985A electrometer. The pyroelectric coefficient was calculated using the following equation:^18^3
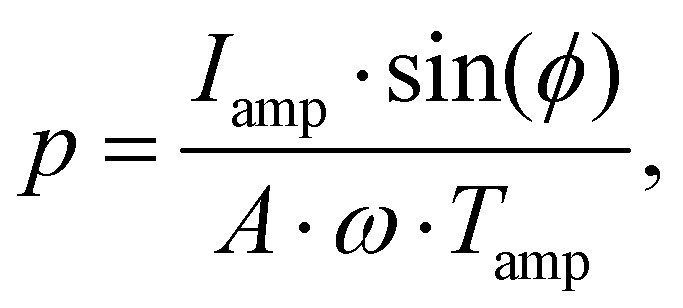
where *I*_amp_ is the amplitude of the measured pyroelectric current, *ϕ* is the phase difference between the temperature and current signal, *A* is the surface area of the electrodes, *ω* is the angular frequency and *T*_amp_ is the amplitude of the temperature modulation, respectively.

## 3 Results and discussion

### 3.1 Differential scanning calorimetry (DSC)

**Fig. 1:**
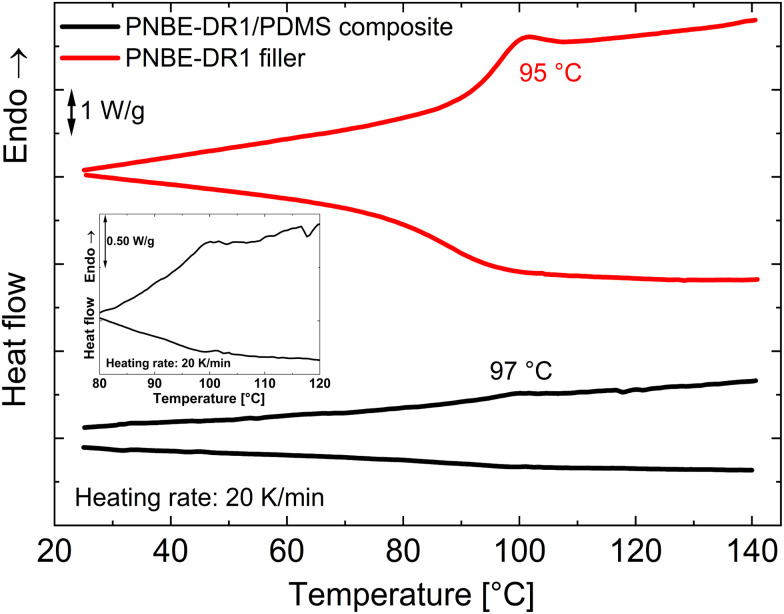
DSC heating (second heating cycle) and cooling (first cooling cycle) thermograms of the neat PNBE-DR1 filler and a 30 wt% PNBE-DR1/PDMS composite. The figure inset shows the magnified thermograms of the composite.

To determine the poling temperature, DSC measurements were performed on a 30 wt% PNBE-DR1/PDMS composite and the corresponding thermograms are shown in Fig. 1 along with those of the neat PNBE-DR1 filler. As seen from the figure, the filler shows a glass transition step during the heating cycle between 90 and 100 °C with a calculated value of 95 °C (*T*_g_). The step is slightly shifted to a lower temperature range on cooling. As expected, during heating, the composite shows a *T*_g_ around 97 °C corresponding to the filler particles (seen more clearly in the inset of Fig. 1). Hence, a temperature of 110 °C—higher than the *T*_g_ of the filler particles was chosen for poling the composites.

### 3.2 Dielectric relaxation spectroscopy (DRS)

**Fig. 2:**
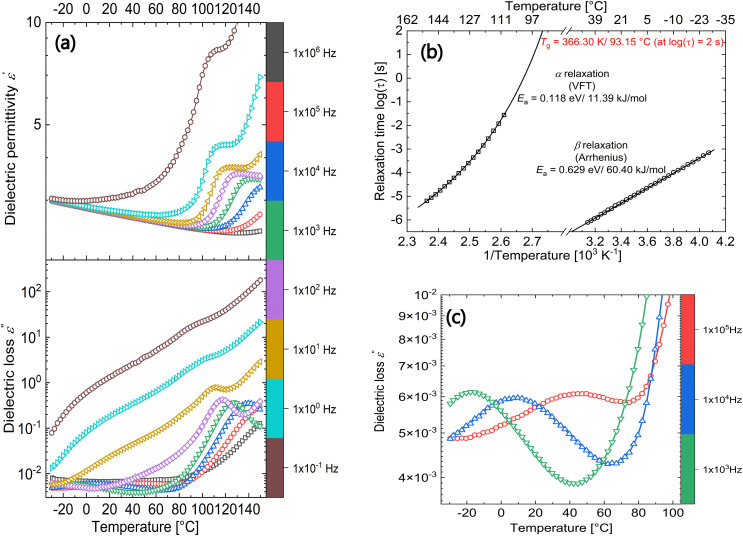
DRS results on a 30 wt% PNBE-DR1/PDMS composite: (a) permittivity and dielectric loss as a function of temperature at selected frequencies from 10^−1^ to 10^+6^ Hz. (b) Arrhenius plot of the observed relaxations. (c) Dielectric loss of the composite between −30 and +100 °C at selected high frequencies.

DRS is a powerful tool to look into the various relaxation processes occurring in the composite under investigation. Fig. 2(a) shows the real (*ε*′) and imaginary (*ε*′′) parts of permittivity plotted as a function of temperature at fixed frequencies from 10^−1^ to 10^+6^ Hz. At negative temperatures below 0 °C, the composite shows a permittivity around 3 and except for the curve measured at 10^−1^ Hz, the value slightly decreases for all other frequencies with an increase in temperature up to 80 °C. As inferred from DSC heating curves, the composite is below the *T*_g_ of the polar filler particles and hence, the filler particles are frozen, leading to low permittivity values observed in this temperature range. However, above 80 °C, there is a sharp increase in permittivity owing to the *T*_g_ of the functionalized polynorbornene filler.

In the dielectric loss plot, corresponding peaks are observed around 100 °C at 10 Hz, which shift to higher temperatures with an increase in frequency as expected from a relaxation process (*α* relaxation).^19^ By fitting the loss peaks (as a function of frequency at different temperatures) with the Havriliak and Negami (HN) function,^20^ relaxation times were extracted. The distribution of the relaxation times was plotted as an Arrhenius plot in Fig. 2b. For the dielectric loss process under consideration a Vogel–Fulcher–Tammann (VFT) fit is obtained indicating a glass-transition relaxation. At a relaxation time of 100 s (log(*τ*) = 2 s), a *T*_g_ of 93.15 °C is obtained,^21^ which agrees very well with the value obtained from DSC. Above the glass-transition relaxation, there is a steep increase in both the permittivity and loss values denoting the increased ionic conductivity in the composite due to the mobile filler chains.

**Fig. 3:**
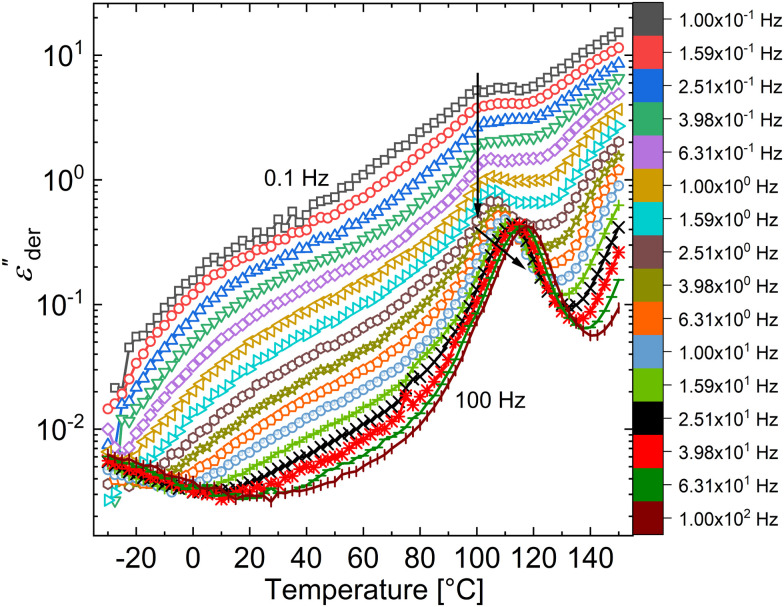
Conduction-free loss 
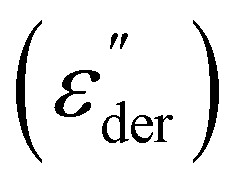
-*versus*-temperature for selected frequencies between 10^−1^ and 10^+2^ Hz in a 30 wt% PNBE-DR1/PDMS composite.

From the dielectric loss plot, two more processes can be identified at lower temperatures below the glass transition. At low frequencies (below 10 Hz), a broad shoulder is observed between −20 and +50 °C and at higher frequencies, an additional relaxation process is observed in the same temperature range (seen more clearly in Fig. 2c). A HN fit of the latter relaxation peaks shows Arrhenius behaviour with an activation energy of 60.40 kJ mol^−1^ (Fig. 2b).^9^ This relaxation can be associated with the limited movement of the polar side groups in the PNBE-DR1 filler particles (*β* relaxation) below its *T*_g_. To have a detailed observation of the former (low frequency) process, the ohmic conduction suppressed loss derivative curves 
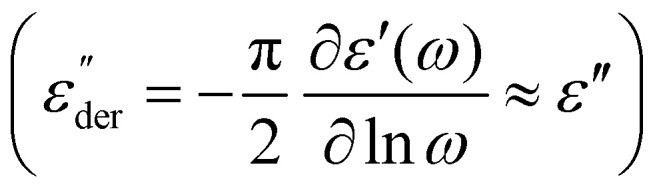
^22,23^ at selected lower frequencies are plotted in Fig. 3.

Looking at Fig. 3, we can observe that the position of the low-temperature shoulder does not change with frequency. Further, on a closer look at *α* relaxation transition, the loss peak centered around 105 °C at 0.1 Hz shows frequency-independent behaviour until about 2 Hz. This suggests an additional process occurring in the glass-transition temperature range of the filler. Though PDMS otherwise considered as an amorphous polymer can form crystals, they melt at a much lower temperature below 0 °C.^24,25^ Similarly, other phase transitions commonly observed in crystalline materials that exhibit such frequency-independent loss peaks can be excluded in the composite.^19^ Hence, the origin of this transition could be due to space-charge-related relaxations that usually occur at low frequencies.^26^

### 3.3 Thermally stimulated depolarization currents (TSDCs)

**Fig. 4:**
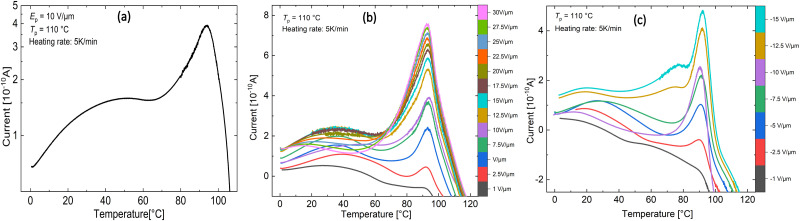
TSDCs measurements on a 30 wt% PNBE-DR1/PDMS composite poled at a temperature (*T*_p_) of 110 °C for a period (*t*_p_) of 10 min (a) with an electric field (*E*_p_) of 10 V μm^−1^, (b) with different positive fields from 1 to 30 V μm^−1^ and (c) with different negative fields from −1 to −15 V μm^−1^.

TSDC technique can be used to complement the results from DRS. From the TSDC curve of the composite under investigation displayed in Fig. 4a, two major processes can be identified. The first peak at 93 °C can be easily identified with the *T*_g_ process. Since the test frequencies (heating rate) employed in TSDC are roughly equivalent to very low frequencies in the range of 10^−3^ and 10^−4^ in DRS,^27^ the second broad shoulder between 30 and 60 °C should have the same origins as that of the low-frequency shoulder observed at low temperatures in the dielectric loss plot (Fig. 2a). To investigate further the origin of this transition, TSDC measurements were performed at different electric fields from 1 to 30 V μm^−1^ and the results are shown in Fig. 4b. While the peak amplitude of the glass-transition peak keeps increasing with the applied electric field, the corresponding amplitude of the low-temperature shoulder saturates above 10 V μm^−1^. In addition, the shoulder position changes with the applied field and shows no trend. These characteristics point the origin of this peak to real charges and hence do not have dipolar origins.^28–30^ The glass-transition peak at higher poling voltages is broad, hinting at the possibility of an additional process. This is confirmed by the presence of a new shoulder below the *T*_g_ peak in Fig. 4c at higher electric fields when poled with a negative voltage.

From previous TSDCs measurements on a pure PNBE-DR1 filler poled at 110 °C published elsewhere,^9^ we can observe that both the shoulder between 20 and 60 °C and the shoulder near the *T*_g_ peak are missing (*cf.*Fig. 4(d) in the literature). Hence, it can be inferred that these processes only present in the composite to be a result of Maxwell–Wagner interfacial (MWI) polarization at the filler-matrix interface.^28,31^ Depolarization of MWI charges in semi-crystalline materials is observed only above their poling temperature.^28,31^ However, here in the case of the composite, since the PDMS matrix is already above its *T*_g_, the onset of *β* and *α* relaxations in the filler could release the trapped interfacial charges leading to the observation of shoulders at their respective temperature ranges in TSDCs. The absence of a clear shoulder in the TSDC measurement reported before between 30 and 60 °C in the composite with a similar 30 wt% filler loading might be due to the low poling field (*E*_p_ = 2 V μm^−1^) used in the work.^9^

### 3.4 Pyroelectric measurements

**Fig. 5:**
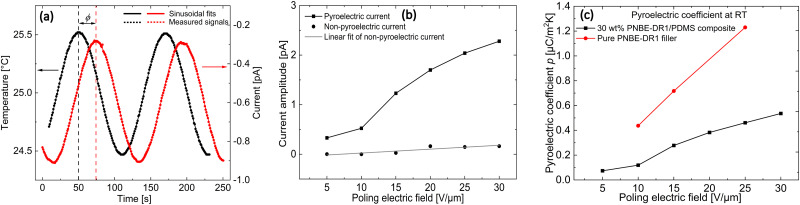
(a) Quasi-static pyroelectric current measurement with low-frequency sinusoidal temperature variation at 25 °C. (b) Individual pyro- and non-pyroelectric components of the measured current from the quasi-static pyroelectric measurements as a function of applied poling field. (c) Pyroelectric coefficient at 25 °C measured on a pure PNBE-DR1 filler and a 30 wt% PNBE-DR1 filled PDMS composite as a function of applied poling field.

As described in Section 2.6, quasi-static pyroelectric measurements were performed at a low frequency of 8.3 mHz on the poled composite. While a phase shift of *ϕ* = 90° is observed for a pure pyroelectric current signal, a non-pyroelectric signal shows 0° phase shift. A phase shift of *ϕ* ≠ 90°, as seen in Fig. 5a, is observed when the total measured current has contributions from both pyroelectric and non-pyroelectric components. Non-pyroelectric components include real charges that can be injected during poling and trapped in the material due to defects, and relaxations or conduction-related processes, contributing to the total measured pyroelectric current.^18^ In case of the composite system under investigation, filler particles containing disperse-red dye or residual ions from solvents, impurities could act as access sites for charge injection. The non-pyroelectric current contribution is excluded in the calculation of the pyroelectric coefficient by using the phase shift angle (sin(*ϕ*)) in eqn (3).^18,32^ By performing a sinusoidal fit of the temperature and current signal, the parameters *T*_amp_ and *i*_amp_ are obtained, which are then inserted in eqn (3) to obtain the pyroelectric coefficient.

Fig. 5b shows the plot containing the pyro-and non-pyroelectric currents calculated using eqn (3). The non-pyroelectric current shows a linear response with increasing poling field as expected, while the pyroelectric current clearly shows a non-linear behaviour with signs of saturation even at low applied poling fields. Accordingly, the calculated pyroelectric *p* coefficient plotted in Fig. 5c shows a non-linear dependence with the poling field. In amorphous glassy polymers, Mospik and Broadhust^33^ proposed that below their *T*_g_, since the dipoles were frozen, the pyroelectric response should primarily arise due to the changes in dipole density resulting from the thermal expansion and contraction, *i.e.*, secondary pyroelectric effects. In such a scenario, linear relationship is expected between the poling field and the pyroelectric coefficient. Looking at the pyroelectric response of the pure polar amorphous filler in Fig. 5c, we indeed observe the same. At 25 °C, similar linear dependency is expected for the composite system under consideration where the polar filler is at a temperature very much lower than its corresponding *T*_g_. In the case of the PDMS matrix, though the measurement temperature is above its *T*_g_, it is non-polar and hence is not expected to contribute to any pyroelectric activity. Considering the above, a non-linear poling field dependency of the *p* coefficient suggests the possibility of a primary pyroelectric effect in the composite, at least at the first glance.

Table 1 lists the *p* coefficients (measured in a quasi-static mode) of the pure PNBE-DR1 filler and a 30 wt% filled PDMS composite at RT compared with a few other polar amorphous polymers reported in the literature. From the table, we can see that the composite shows a maximum *p* coefficient of 0.54 μC m^−2^ K^−1^ when poled with 30 V μm^−1^ and the pure polar amorphous filler shows a higher value of 1.23 μC m^−2^ K^−1^ for a lower poling field. While the *p* coefficient of the pure filler is comparable to the other similar polar amorphous polymers reported before, the composite shows a lower value as expected due to the lower filling load. Since 30 wt% filler loading was found to be more homogenous than the other compositions,^9^ the pyroelectric measurements were limited to only these samples. Despite the lower *p* coefficient value, the elasticity of the composite offers additional advantages over glassy polymers to be used in stretchable applications.

**Table 1:** Quasi-static pyroelectric *p* coefficient at RT poled with a field *E*_p_ for a 30 wt% PNBE-DR1/PDMS composite compared with other polar amorphous polymers from the literature

Polymer	*p* [μC m^−2^ K^−1^]	*E*_p_ [V μm^−1^]	Ref.
30 wt% PNBE-DR1/PDMS composite	0.46	25	This work
	0.54	30	This work
PNBE-DR1 filler	1.23	25	This work
Azobenzene alkoxy-substituted polyvinyl alcohol	0.24	25	34
Polyacrylonitrile-*co*-vinylacetate	1.94	30	35
Nitroaniline-substituted thermoplastic polyurethane	1.3	30	36

### 3.5 TSDC cycling and partial depolarization experiments

**Fig. 6:**
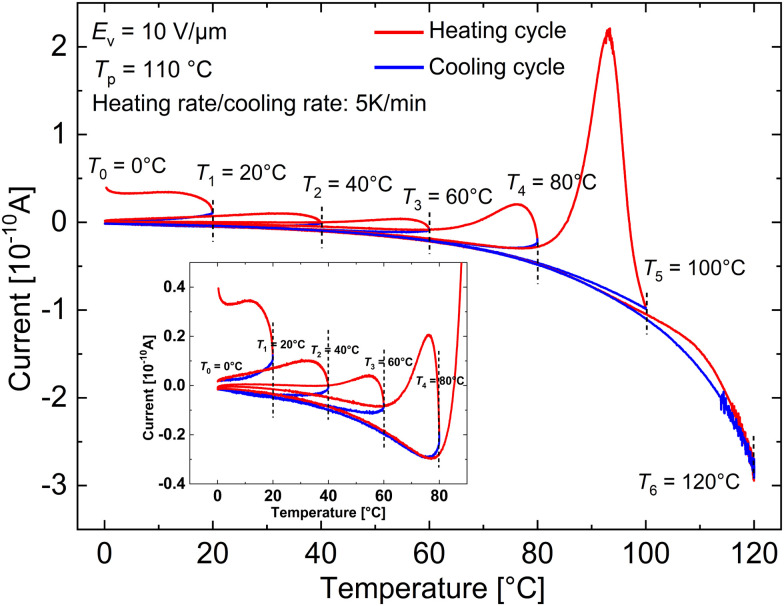
Cyclic TSDC measurements where a 30 wt% PNBE-DR1/PDMS composite film after initially cooled down to 0 °C under *E*_p_ = 10 V μm^−1^ from 110 °C is heated to a temperature *T*_max_ (from 20 to 120 °C in 20 °C steps). At the end of each cycle, the sample was immediately cooled from *T*_max_ to 0 °C before starting the next cycle. The inset figure shows the enlarged plot of the initial four cycles.

Looking at the results from TSDC measurements, it is clear that pyroelectric activity at RT is influenced by real charges. At the same time, the field dependency of pyroelectric *p* coefficient shows a non-linear behaviour expected from the pyroelectric effect dominated by the change in spontaneous polarization of the dipoles (primary pyroelectricity), commonly observed in ceramics and crystalline polymers.^37^ To gain more insights into the pyroelectric activity of a 30 wt% PNBE-DR1/PDMS composite and determine its temperature dependence, cyclic depolarization measurements were made on the sample as previously employed by DeRossi *et al.*^38^ Here the composite film was poled at 110 °C with a field of 10 V μm^−1^ for 10 min before cooling down to 0 °C with the field—typical poling procedure prior to a TSDC measurement. After removing the field, the sample was partially depolarized by heating it up to 20 °C (*T*_max_) and then cooling it back to 0 °C. This was followed by heating the sample again to a new *T*_max_ that was 20 °C higher than the previous cycle, *i.e.*, 40 °C. The measurement was repeated until a *T*_max_ = 120 °C was reached. The depolarization currents are measured both during the heating and cooling cycles and are plotted in Fig. 6.

The observations from each cycle are listed below:

• Cycle *T*_1_ = 20 °C: on cooling from 20 °C, the current is significantly lower than that during the initial heating, indicating the release of non-dipolar charges when the sample is first heated to 20 °C.

• Cycle *T*_2_ = 40 °C: the contribution to the total measured current due to MWI polarization-related charges can be observed due to the increasing current in this temperature range leading to a peak just before 40 °C (more clearly seen in the inset of Fig. 6). This is also inferred by the decrease in the total heating and cooling current in this cycle compared to the corresponding previous cycle.

• Cycle *T*_3_ = 60 °C: as the sample is heated to a higher temperature, we observe that the heating current is constant and comparable to the previous cooling curve until the previous *T*_max_ = 40 °C. This shows that the pyroelectric effect observed in this temperature range is stable and, in the absence of MWI polarization, has contributions primarily due to the change in thermal expansion of the composite system, *i.e.*, secondary pyroelectricity. The change in slope of the current above 40 °C and its subsequent decrease during the cooling cycle indicates that MWI polarization charges are still present in the sample. However, after heating the sample to 60 °C, we can observe that the cooling current follows the same trend as the previous cooling cycle, indicating that the majority of the non-dipolar charges contributing to the TSDC shoulder observed between 30 and 60 °C in Fig. 4a are released at the end of this cycle.

• Cycle *T*_4_ = 80 °C: once again, similar values of the present cycle's heating current and the previous cycle's cooling current until the previous cycle's *T*_max_ = 60 °C show that the thermal expansion and contraction in the composite are uniform in this temperature range and a stable pyroelectric effect follows suit. On continuing to heat the sample to 80 °C, we observe the release of MWI polarization charges that are observed in the form of a shoulder in the similar temperature range in TSDC (Fig. 4c) due to the onset of thermal motion in the backbone of filler particles. This results in a steep increase in the depolarization current above 60 °C, higher than that observed in the last two heating cycles.

• Cycle *T*_5_ = 100 °C: the heating current remains essentially the same as the previous cooling cycle until the previous *T*_max_, followed by a large increase in the depolarization current as the sample passes through the glass-transition temperature of the filler particles.

• Cycle *T*_6_ = 120 °C: from the final heating and cooling cycle that coincides with the previous cooling cycle, we can infer that all the spontaneous or induced polarization in the PNBE-DR1 polar filler particle is lost at the end of the previous heating cycle as expected, resulting in the observation of a baseline TSDC current.

To summarize the important observations of the cyclic measurements in Fig. 6, the pyroelectric current in the composite has clear contributions from at least two processes. The first is MWI polarization due to non-dipolar charges trapped in the filler-matrix interface. The onset of *β* transition (restricted movement of polar side groups) below RT triggers the continuous release of some of these charges, evident from the subsequent decrease of heating current in the first three cycles (until *T*_max_ = 60 °C). At higher temperatures, near the *T*_g_ of the filler, the remaining real/space charges are released from the interface owing to the increased thermal motion of the filler (Cycle *T*_4_). The second process is the change in dipole density of the composite due to thermal expansion and contraction, resulting in a secondary pyroelectric effect. This can be inferred from the heating curves of cycles *T*_1_ to *T*_5_, which follow their corresponding previous cooling curve until the temperature reaches the previous *T*_max_.^38^ This observation is possible due to the removal of non-dipolar charges in the measured temperature range during the previous heating cycle. Hence, the secondary pyroelectric current is stable and reversible until 80 °C. Finally, during the glass transition, all the polarization in the filler induced due to the poling process is lost, giving rise to a baseline TSDC current in cycle *T*_6_.

The real charges at the interface of two different phases that give rise to MWI polarization in semi-crystalline and other inhomogeneous materials, such as composites, have been shown to contribute to the pyroelectric effect.^31,34,39,40^ The distance between these MWI charges with opposite signs can vary due to the difference in the thermal expansion coefficient between the different phases contributing to a pyroelectric current. This additional contribution of MWI polarization to the total measured pyroelectric current and its field-dependent increase with increasing field (attributed to an increase in charge mobility) can also explain the non-linear behaviour of the pyroelectric coefficient with the poling field.^31,34^

**Fig. 7:**
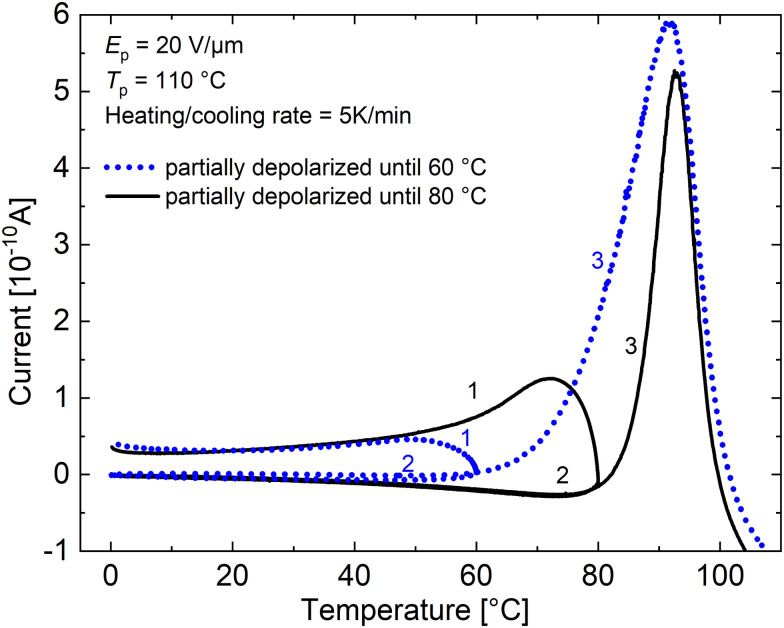
TSDC partial depolarization experiments on a 30 wt% PNBE-DR1/PDMS composite film with *E*_p_ = 20 V μm^−1^ and *T*_p_ = 110 °C. After poling, the sample was cooled to 0 °C with the field and then partially depolarized up to 60 or 80 °C. This was followed by again cooling the sample back to 0 °C before heating it to 130 °C. The numbers near the curves denote the order in which the TSDC measurement was carried out.

By heating and partially depolarizing the sample to 80 °C after a typical TSDC measurement, MWI charges can be removed from the composite and a subsequent pyroelectric measurement at RT should have contribution only from dipole-related processes. This can be observed in Fig. 7 (solid line in black), where both the MWI shoulders appearing below the *T*_g_ in Fig. 4 are removed after initially heating the sample to 80 °C. Hence, the current observed during cooling of the sample from 80 to 0 °C coincides with the subsequent heating of the sample in the same temperature range. In case of the sample partially depolarized until 60 °C (dotted line in blue), though the low temperature shoulder is removed during the initial heating procedure, we still observe the release of MWI charges above 60 °C, evident by the broad shoulder around 80 °C and higher peak amplitude of the glass-transition relaxation. Thus, it is necessary to depolarize the sample until 80 °C to remove the MWI polarization contribution to the total observed pyroelectric current.

It is possible to calculate the pyroelectric *p* coefficient from TSDC measurements as well using the below equation:^18^4
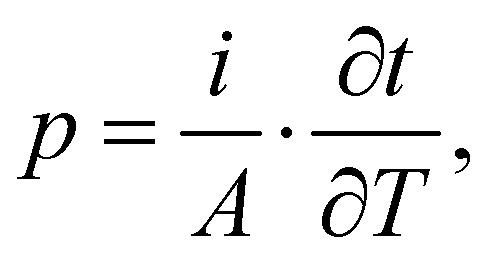
where *i* is the measured depolarization current at a given temperature and ∂*T*/∂*t* is the heating rate used during the depolarization step of TSDC. Similar to the case of the periodic temperature variation method introduced earlier in this work, the measured current has a contribution from non-dipolar processes. The currents rising from these real charges are superimposed and cannot be separated using this method.^18^ Hence, the calculated *p* coefficient using TSDC technique is higher than that calculated using the quasi-static periodic temperature variation method, where using eqn (3), it is possible to separate the non-pyroelectric contributions.^40^

While measuring the *p* coefficient using the temperature variation method, a value of 0.38 μC m^−2^ K^−1^ was obtained at 25 °C (*cf.*Fig. 5c) and a value of 2.26 μC m^−2^ K^−1^ was calculated using eqn (4) for the TSDC method (*E*_p_ = 20 V μm^−1^ and *t*_p_ = 10 min). However, by employing the partial depolarization procedure, we now know that the non-pyroelectric contributions can be separated, ideally yielding a similar *p* coefficient using both methods. To verify this, the pyroelectricity was measured in the sample after partially depolarizing it until 80 °C (*E*_p_ = 20 V μm^−1^ and *t*_p_ = 10 min). Similar values of 0.05 μC m^−2^ K^−1^ and 0.07 μC m^−2^ K^−1^ were computed from the temperature variation and TSDC method, respectively. From this, we can once again confirm that the measured pyroelectric current after cleaning at 80 °C has only dipolar origins. Comparing with the total *p* value (0.38 μC m^−2^ K^−1^) measured in the neat sample (without partial depolarization), we find it is an order of magnitude lower. Though some dipoles may be depolarized, while initially heating to 80 °C, still majority of the dipoles should be frozen below *T*_g_. Hence, the difference in the pyroelectric current measured in the neat sample should arise from MWI polarization. Since the filler particles are rigid at RT, MWI charges give rise to a stable and persistent pyroelectric current.^34^

As *α* relaxation has the highest strength due to the movement of the whole polymer backbone (filler particles), more MWI charges are released during the onset of this process. This can be observed in Fig. 7 from the shoulder of the *T*_g_ peak in the composite sample after it was partially depolarized up to 60 °C (dotted blue line ‘3’). After the partial depolarization procedure, a quasi-static pyroelectric measurement at RT yielded a *p* = 0.11 μC m^−2^ K^−1^, closer to the total *p* coefficient of 0.38 μC m^−2^ K^−1^ measured initially before cleaning. This shows that though some of the pyroelectric activity is lost after the sample is heated above RT due to *β* relaxation, the *p* coefficient in the composite is more or less stable until the onset of *T*_g_ relaxation.

### 3.6 Estimation of spontaneous polarization in a 30 wt% PNBE-DR1/PDMS composite

As the dipoles are frozen below the *T*_g_ of the filler, it is not possible to determine the polarization at RT by measuring the dielectric hysteresis.^41,42^ Since charges can be injected into the sample by applying an electric field near or above the *T*_g_ where it is possible to induce a polarization in the sample, an accurate determination of the polarization induced in the filler is also not possible. However, by partially depolarizing the sample until 80 °C, the MWI polarization charges from a 30 wt% PNBE-DR1/PDMS composite are removed, resulting in only a glass-transition peak observed during the subsequent TSDC measurement (Fig. 7, solid black line ‘3’). Hence, by integrating the area under the peak, the spontaneous polarization in the sample can be obtained for a particular *E*_p_.^28^

**Fig. 8:**
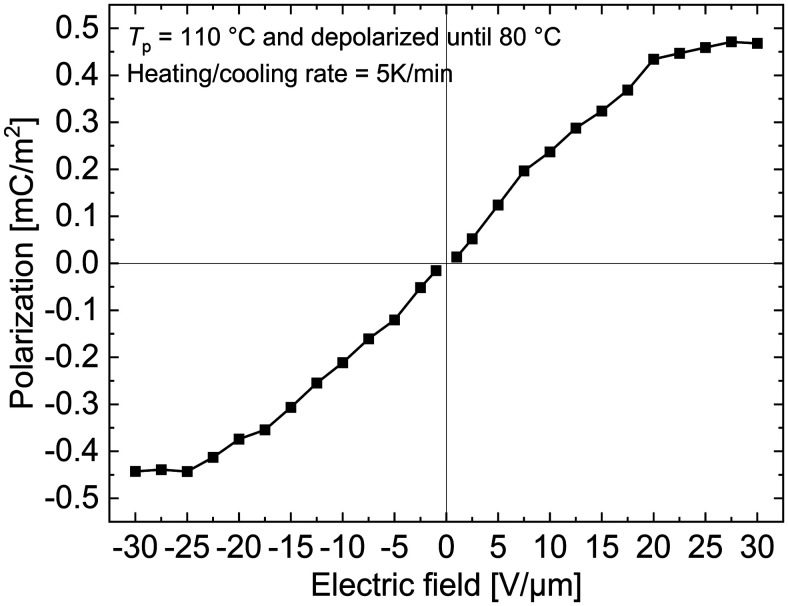
Polarization-*versus*-electric field curve of a 30 wt% PNBE-DR1/PDMS composite calculated from the area of the glass transition peak after the sample was partially depolarized until 80 °C.

Fig. 8 depicts the polarization calculated using the integral approach for different poling fields in positive and negative directions until ±30 V μm^−1^. The polarization initially shows linear behaviour with the applied field, but soon saturates at fields above ±20 V μm^−1^. This can be understood as a result of the low filler loading (30 wt%). Theoretically, one can obtain the polarization value from the dielectric constant and the applied field using eqn (5):

5 *P* = *ε*_0_(*ε*′ − 1)*E*,

where *ε*_0_ is the permittivity of free space (8.54 × 10^−12^ F m^−1^), *ε*′ is permittivity of the sample and *E* is the applied electric field. Using a measured permittivity value of 3.5 (at 1 kHz and 150 °C in Fig. 2a) and an electric field of 5 V μm^−1^, a theoretical polarization of 0.11 mC m^−2^ is calculated. From Fig. 8, a similar value of 0.12 mC m^−2^ is obtained for the same poling field. For *E* = 20 V μm^−1^ above which the polarization saturates, a theoretical value of 0.44 mC m^−2^ is estimated, while from partially depolarized TSDC measurements a value of 0.43 mC m^−2^ can be calculated. Hence, using the TSDC method, it would be possible to measure the induced polarization in the sample. Finally, by correlating the polarization behaviour seen in Fig. 8 with the saturation of the overall TSDC curves (having contribution from both dipoles and MWI polarization) in Fig. 4b and c at higher poling fields, we can explain the tendency of *p* coefficient^7^ approaching a plateau in the composite at these electric fields (Fig. 5c).

## 4 Summary and conclusions

In the current study, the pyroelectric properties of a poled stretchable electret composite filled with 30 wt% of a functionalized polar polynorbornene polymer (PNBE-DR1) in a polydimethylsiloxane (PDMS) matrix were investigated and correlated with the results obtained from differential scanning calorimetry (DSC), dielectric relaxation spectroscopy (DRS) and thermally stimulated depolarization current (TSDC) technique.

From the investigation of the dielectric properties of the composite, an *α* relaxation corresponding to the glass transition (around 95 °C) and a *β* relaxation (around RT) associated with the movement of small side groups in the polar filler are observed. On the other hand, TSDC measurements reveal that real charges trapped at the filler-matrix interface are released on the onset of *α* and *β* relaxations, leading to the observation of Maxwell–Wagner interface (MWI) polarization shoulders.

Cyclic TSDC measurements were made where the sample after the poling step was selectively depolarized in 20 °C steps from 0 °C to 120 °C, followed by cooling the sample back to 0 °C at the end of each cycle. The continuous decrease of the heating currents in the first three cycles until the samples was depolarized to 60 °C and its subsequent increase in the fourth cycle clearly show that non-dipolar charges are continuously released as the sample is heated from 0 °C until the *T*_g_ of the filler. The convolution of the cooling cycles and the tendency of the heating currents at higher temperatures to follow the previous cooling cycle point out the stable contribution from secondary pyroelectricity due to a change in thermal expansion/contraction (change in dipole density) in this temperature range.

The pyroelectric *p* coefficient calculated at RT from the TSDC measurement after partial depolarization up to 80 °C is similar to that calculated using the quasi-static periodic temperature variation method. On the other hand, the estimated values are one order of magnitude lower than those obtained without the partial depolarization. This shows that it is possible to separate the MWI contribution from the total measured pyroelectric current and the maximum contribution to pyroelectricity at RT comes from it. The *p* value at RT measured using the temperature variation method after partial depolarization up to 60 °C is similar to that calculated in a neat sample, inferring that major MWI depolarization takes place near the glass-transition temperature. Hence, the pyroelectric current due to MWI polarization is stable at RT though we observe a small hysteresis in the heating and cooling currents during the cyclic measurement from 20 °C to 40 °C. The results of cyclic and partial TSDC measurements show that the *p* coefficient is stable in the temperature range from 0 °C to 80 °C.

The pyroelectric *p* coefficient estimated using the quasi-static periodic temperature variation shows a non-linear increase with the applied electric field and signs of saturation at higher electric fields. Since primary pyroelectricity due to a change in spontaneous polarization is not expected in amorphous materials, the observed non-linearity should arise from the change in charge mobility of MWI charges at higher electric fields. The saturation of MWI shoulders as well as spontaneous polarization at relatively low applied fields support the plateauing of *p* coefficient curve at these fields. A maximum *p* co-efficient of 0.54 μC m^−2^ K^−1^ is estimated for an applied electric field of 30 V μm^−1^. Further, the role of non-dipolar MWI polarization in contributing to the piezo- and pyro-electric currents measured at RT is clearly shown. A thorough dielectric investigation enables the separation of MWI and dipolar charges using the TSDC technique, thus, making it possible to determine the spontaneous polarization *vs.* electric field behaviour of the composite. The calculated polarization values agree very well with those theoretically estimated.

The results of this study shed light into the mechanisms responsible for the observed electro-active properties in a novel all-organic polar amorphous composite. This in turn will help in improving, tailoring and developing the piezo- and pyro-electric properties of such fluorine-free materials, which are the need of the hour. The following approaches to improve the overall pyroelectric coefficient of such composites can be explored in the future:

• Replacing the PDMS matrix with a polymer possessing a higher coefficient of thermal expansion.

• Decreasing the filler particle size or increasing the filler loading will cause an overall increase in the interfacial area resulting in an increased MWI polarization.

• Increasing the dielectric permittivity of the filler particles will result in a higher degree of polarization, which can be frozen during the poling step. This, in turn, can lead to a higher pyroelectric current due to the secondary pyroelectric effect.

• Introducing a crystalline or weakly crystalline filler will lead to additional pyroelectricity from the change in the dipole moment of the filler (primary pyroelectricity).

## Author contributions

R. V. T designed and performed the experiments, formally analyzed the results, and wrote the original manuscript. D. M. O initiated the activity, designed the materials, acquired funding and supervised this research. F. O. synthesized the filler and prepared the composite films. M. S. wrote the program for TSDC and pyroelectric measurements, and offered technical help. All authors contributed to discussions, reviewing, and editing, and approved the final version of the manuscript.

## Conflicts of interest

There are no conflicts to declare.

## Supplementary Material
